# Community and motivation among tennis officials: a cross-cultural multilevel analysis

**DOI:** 10.3389/fpsyg.2023.1238153

**Published:** 2023-12-19

**Authors:** Longxi Li, Yanfeng Li

**Affiliations:** ^1^Center for Leadership in Athletics, University of Washington, Seattle, WA, United States; ^2^Hebei Institute of Physical Education, Shijiazhuang, Hebei, China

**Keywords:** sport, officiating, scale validation, satisfaction, hierarchical model

## Abstract

Sport officials are pivotal to the development of the game at every level. Yet, the exploration of these officials’ job satisfaction and turnover intentions, especially within tennis, remains largely neglected. This study undertakes a cross-cultural adaptation and validation of the Referee Retention Scale (RRS) in a Chinese context (RRS-CN) and uses multilevel models (MLM) to explore the influence of perceived administrator consideration, mentoring, continuing education opportunities, remuneration, stress, and ecological factors on tennis officials’ sense of community and officiating motivation. Data from 523 tennis officials across 26 provinces in China were gathered via an online survey. Through exploratory and confirmatory factor analyses, the RRS-CN was validated as a culturally adaptive 25-item scale. In the following, MLM results revealed that officiating levels, socioeconomic status, perceived administrator consideration, mentoring, and levels of continuing education significantly predict officials’ sense of community. Additionally, we identified that continuing education, mentoring, and remuneration significantly influences officiating motivation. These findings underscore the importance of fair assignments, mentorship, and ongoing professional development in enhancing job satisfaction and retention. Future explorations are encouraged to extend the analysis to more ecological variables and further investigate their potential effects on systematic partial nesting, enhancing the generalizability and precision of measurement in job satisfaction and turnover studies across diverse cultural landscapes.

## Community and motivation among tennis officials: a cross-cultural multilevel analysis

Organized sports are heavily reliant on officials; however, the number of qualified personnel continues to decline, posing a significant challenge to the sports industry (Kim, 2016; Ridinger et al., 2017). The shortage of officials in various sports can negatively impact both the quantity and quality of game competitions and spectator experiences. In many instances, games must be rescheduled or canceled due to the unavailability of officials (Zvosec et al., 2021). Overworked official teams and inexperienced officials thrust into situations beyond their skill level contribute to a toxic environment within the industry (Read, 2000; Cuskelly and Hoye, 2004). Previous research has highlighted the increasing difficulty of recruiting new referees, necessitating actions to expand the officiating community (Ridinger et al., 2017). To more effectively recruit and retain sports officials, it is crucial for sports administrators to understand the factors influencing job satisfaction (Warner et al., 2013; Ridinger et al., 2017). A growing body of literature addresses various issues related to this community, examining the entire process from entering to leaving officiating roles (Purdy and Snyder, 1985; Kellett and Shilbury, 2007; Warner et al., 2013; Ridinger, 2015; Livingston et al., 2017; Ridinger et al., 2017). With attrition rates of nearly 30%, sport officials are leaving their positions due to many reasons, for instance, abuse (Radziszewski et al., 2023); lack of respect and recognition (Hancock et al., 2015); and gendered aggressions (Webb et al., 2021; Tingle et al., 2022).

Meanwhile, several contributors were found that should consider ways of enhancing sport officials’ engagement and retention; a sense of community was known as a strong predictor (Zvosec et al., 2021; Kim et al., 2022). Sport brings together people of diverse backgrounds into many common communities where they can feel a sense of belonging (Kellett and Warner, 2011). A community that seems to provide more opportunities for meaningful social interactions has been shown to be important to sport officials where they can feel safety, belonging, and attachment (Kim et al., 2022). A strong sense of community led to greater psychological wellbeing, positive officiating experiences, and better officiating persistence (Zvosec et al., 2021; Kim et al., 2022). In addition, Hancock et al. (2015) demonstrated that sport officials were motivated to begin officiating for intrinsic reasons and cited intrinsic and social motivations for continuing officiating. Furthermore, mentorship, perceived organizational support, and financial remuneration were found as contributors to retaining sport officials (Cuskelly and Hoye, 2013; Kim, 2016; Livingston et al., 2017; Kim et al., 2022).

As vital facilitators of match competitions and integral components of tennis culture, tennis officials play an irreplaceable role in the game (Lake and Osborne, 2019). A tennis official is a person who “helps ensure that any given tennis match is conducted under the fairest possible conditions” (United States Tennis Association, 2016, p. 1). Tennis officials encompass various roles, including referee, chief umpire, chair umpire, and line umpire, with differing responsibilities on and off the court. In Asia, and particularly in China, the tennis community has experienced dramatic growth over the past decade (Rick and Li, 2023). Although tennis is considered a minority sport in China, the country’s large population makes a significant contribution to global participation rates (Rick and Li, 2023). According to the International Tennis Federation (2021), an estimated 20 million Chinese play tennis, almost one-fourth of the world’s total. Meanwhile, the expansion of tennis events in China has been instrumental in the sport’s development (Mangan and Dong, 2009). In collaboration with the Women’s Tennis Association (WTA), Association of Tennis Professionals (ATP), and International Tennis Federation (ITF), China hosted more than 70 international tournaments in 2019, comprising 80% of all professional tennis events held in Asia (General Administration of Sport of China, 2019). This growth in events marks a significant milestone in the development of tennis officials in China. As of 2016, approximately 60 white badge or higher ranking officials, 300 national-level officials,^1^ and thousands of junior-level officials have worked in over 21,000 domestic and international tournaments (Xinhua News Agency, 2016). Despite the growing official community, the number of officials has not grown at the same rate as the number of participants and events in China.

Noteworthily, existing research has primarily examined sports officials within a Western context. Only a limited amount of sport officials related studies were found from Africa (Mkumbuzi et al., 2023); the Middle East (Qader, 2023); and Asia (Kim and Hong, 2016; Kim, 2016). Thus, studies focusing on a globally symbolic sport like tennis, and its rapidly growing officiating community in China, could provide valuable insights for understanding this unique stakeholder group as part of the sport ecosystem and has practical implications related to officiating sense of community and motivation. Additionally, multilevel modeling (MLM) should be employed more frequently in sport management research to ensure appropriate data analysis and interpretation as data are often nested (Swierzy et al., 2019). Consequently, this study considers both individual-level factors and macro-level factors, such as the province where a tennis official is registered, in relation to job satisfaction.

A two-fold study was conducted to address this gap in literature. First, given the absence of relevant measurements in Chinese, we translated the Referee Retention Scale (RRS) developed by Ridinger et al. (2017) and established a validated Chinese version (RRS-CN). The RRS-CN was then utilized in the main study, which aimed to answer two primary research questions within the context of tennis officials in China:

1. To what extent do individual and provincial factors, such as administrator consideration, mentoring, continuing education, remuneration, stress, and the number of events hosted, influence tennis officials’ sense of community in officiating? Are these effects moderated by officials’ officiating level, age, gender, and socioeconomic status (SES)?
2. Furthermore, how do individual and provincial factors, including administrator consideration, mentoring, continuing education, remuneration, stress, and the number of events hosted, affect tennis officials’ motivation to continue officiating? Are these effects moderated by officials’ officiating level, age, gender, and SES?

## Methods

### Participants

Tennis officials (*N* = 523, female = 143, male = 380) were recruited online from 26 provinces in China. Participants were a convenience sample of varying categories of tennis officials included white badge or higher level (*n* = 32), national-level (*n* = 154), and junior-level officials who were not yet promoted to the national level (*n* = 337), albeit with a ratio of official levels mirroring national estimates of the population (Xinhua News Agency, 2016). The majority (95%) of the participants reported bachelor’s or post-graduate degrees, and 39% of them were employees in higher education institutions. Approximately half of the participants were recruited from the central and southeastern coastal regions (see Appendix A Table 1).

### Procedures

Participants were collected in two waves of data collection via an anonymous online survey on Tencent Survey (Tencent Inc.). In November 2019, 31 provincial-level tennis administration agencies were contacted by the corresponding author and 26 of them consented to take part in facilitating the data collection (response rate: 84%). In the first wave of data collection, an online survey link with recruiting message was distributed by provincial-level agencies to individual tennis officials who registered in their provinces via WeChat, a mainstream social media platform in the mainland (Tencent Inc.). The background of the study, purpose, and voluntary participation information were introduced on the first page of the survey. Participants E-signed an online informed consent form before enrollment in the study. In the second wave, a reminder message with the same survey link was sent 2 weeks after the first wave. In total, 1,152 cases were recorded, after the exclusion of incomplete and inconsistent cases, 523 participants remained in the convenience sample (first wave *n* = 180, second wave *n* = 343, overall completed response rate: 45.2%). To note, the first and second wave samples were used in the RRS-CN validation and reliability testing process (see Appendix A), and the combined sample (*n* = 523) was analyzed in the main study. The procedure used in the current study was approved by the institutional ethics committee and adhered to the ethical principles of the Declaration of Helsinki.

### Measures

#### Measurement validation and internal reliability

The RRS was first translated into Chinese simplified by the authors and evaluated by two bilingual expert consultants in a related field (Ridinger et al., 2017; Li et al., 2022). Following the methodological steps described by Mimura and Griffiths (2007), we adopted the forward–backward translation method due to its established efficacy in ensuring fidelity during cross-cultural scale translation. In alignment with these steps, the preliminary RRS-CN, once achieving satisfactory agreement, was translated back to English for a comprehensive accuracy check. This was followed by a re-translation to simplified Chinese. Before commencing data collection, the final RRS-CN was scrutinized by authors and expert consultants to validate its precision and consistency. Demographic data, including geographic location, age, gender, official level, educational background, occupation, and income, were self-reported. An overview of RRS-CN is in Appendix A Table 2.

#### Main study

Participant demographic characteristics questions were identical to the measurement validation sample, and all information was self-reported. Participants’ socioeconomic status (SES) scores were first calculated based on income, educational background, and occupation responses and then categorized into three levels, high, medium, and low (Chen et al., 2018; Wani, 2019). Events hosted data were calculated based on the 2019 China Tennis Association event schedule (General Administration of Sport of China, 2019). Two outcome variables, sense of community and intrinsic motives (hereafter motivation) related to officiating, were measured using three and six Likert items on the RRS-CN, respectively. Five level 1 predictors, administrator consideration, mentoring, continuing education, lack of stress (hereafter stress), and remuneration, were measured using five subscales of RRS-CN, respectively. Participants indicated their degree of agreement on a 7-point Likert scale from 1 (strongly disagree) to 7 (strongly agree) where RRS-CN applied. Unweighted average scores of each outcome variable and predictor were calculated for data analyses. Internal consistency of the measurement was evaluated through Cronbach’s alpha (in the current study, *α* ranged from 0.72 to 0.92. which were consistently above the acceptable threshold of 0.70, see Appendix Table 2). Furthermore, an overview of the predictors and outcome variables in the main study is illustrated in Table 1.

**Table 1:** Overview of variables.

Variable name	Brief description	Predictor level	Analytic function
L2_Site	Provinces identifier where tennis officials were recruited from: 1, 2, 3, …, 26	Level 2	
L1_ID	Participant identifier ID: 1, 2, 3, …, 523	Level 1	
			
Community	*Sense of community*	Level 1	Outcome
	Rating scale (1 = strongly disagree; 7 = strongly agree); average score of three Likert items in RRS-CN (V18-V20);		
	Brief definition^a^: Perceived sense of belonging to a supportive community of officials		
Motivation	*Intrinsic motives*	Level 1	Outcome
	Rating scale (1 = strongly disagree; 7 = strongly agree); average score of six Likert items in RRS-CN (V01-V06);		
	Brief definition: Reasons related to enjoyment of competition and staying involved with a sport that attract someone to the role of officiating		
FemEff	Effect-coded female status, 1 = female, −1 = male	Level 1	Predictor
L2_Fem	Level 2 standardized mean province-level female officials recruited aggregate, grand-mean centered, in *SD*s	Level 2	Predictor
Off_Level_W	Effect-coded referee level, international level (white badge and/or higher) = 1, lower than national level = −1, else = 0	Level 1	Predictor
L2_Off_Level_W	Level 2 standardized mean province-level white-card level officials recruited aggregate, grand-mean centered, in *SD*s	Level 2	Predictor
Level_N	Effect-coded referee level, national level = 1, lower than national level = −1, else = 0	Level 1	Predictor
L2_Off_Level_N	Level 2 standardized mean province-level national-level officials recruited aggregate, grand-mean centered, in *SD*s	Level 2	Predictor
Age	Effect-coded age, 1 = 30 or younger (yrs), −1 = above 30 (yrs)	Level 1	Predictor
L2_Age	Level 2 standardized mean province-level officials age 30 and younger recruited aggregate, grand-mean centered, in *SD*s	Level 2	Predictor
SES_H	Effect-coded socioeconomic status, high category 1 = 1, middle category 2 = −1, else = 0	Level 1	Predictor
L2_SES_H	Level 2 standardized mean province-level upper-level socioeconomic status recruited aggregate, grand-mean centered, in *SD*s	Level 2	Predictor
SES_L	Effect-coded socioeconomic status, low category 3 = 1, middle category 2 = −1, else = 0	Level 1	Predictor
L2_SES_L	Level 2 standardized mean province-level lower-level socioeconomic status recruited aggregate, grand-mean centered, in *SD*s	Level 2	Predictor
Event_Eff	The total amount of national and/or international tennis events hosted in provinces, one or more events hosted category 1 = 1, no event hosted category 0 = −1	Level 2	Predictor
Admin	*Administrator consideration*	Level 1	Predictor
	Rating scale (1 = strongly disagree; 7 = strongly agree); average score of three Likert items in RRS-CN (V25, V27, V28);		
	Brief definition: Level of perceived fairness and consideration from assigners and administrators		
L2_Admin	Level 2 standardized mean province-level administrator consideration aggregate, grand-mean centered, in *SD*s	Level 2	Predictor
Stress	*Lack of stress*	Level 1	Predictor
	Rating scale (1 = strongly disagree; 7 = strongly agree); average score of three Likert items in RRS-CN (V15-V17);		
	Brief definition: Infrequent encounters with stressful situations related to officiating		
L2_Stress	Level 2 standardized mean province-level lack of stress aggregate, grand-mean centered, in *SD*s	Level 2	Predictor
ConEducation	*Continuing education*	Level 1	Predictor
	Rating scale (1 = strongly disagree; 7 = strongly agree); average score of three Likert items in RRS-CN (V21-V23);		
	Brief definition: Preparation from ongoing education and training to deal with various aspects of officiating		
L2_ConEducation	Level 2 standardized mean province-level continuing education aggregate, grand-mean centered, in *SD*s	Level 2	Predictor
Mentoring	*Mentoring*	Level 1	Predictor
	Rating scale (1 = strongly disagree; 7 = strongly agree); average score of four Likert items in RRS-CN (V11-V14);		
	Brief definition: Support and encouragement from a mentor or a friend to become involved with officiating		
L2_Mentoring	Level 2 standardized mean province-level mentoring aggregate, grand-mean centered, in *SD*s	Level 2	Predictor
Remuneration	*Remuneration*	Level 1	Predictor
	Rating scale (1 = strongly disagree; 7 = strongly agree); average score of three Likert items in RRS-CN (V07, V09, V10);		
	Brief definition: Financial payment for officiating sporting events		
L2_Remuneration	Level 2 standardized mean province-level remuneration aggregate, grand-mean centered, in *SD*s	Level 2	Predictor

*N* = 523 participants within 26 provinces; event data were extracted from the China Tennis Association (CTA) 2019 event schedule.

a Brief definitions of level 1 psychometrical predictors were extracted from Table 2 (p. 518) from Ridinger et al. (2017); to note, V01-V28 (except V08, V24, AND V26) were used in the main study, and items are illustrated in Appendix A Table 2.

### Data analysis

#### Measurement validation and internal reliability

To validate RRS-CN, an exploratory factor analysis (EFA) was used to provide initial internal structural evidence for the items. EFA was based on the first wave of collected data. In the following, to verify the factor structure extracted from the EFA, we conducted a confirmatory factor analysis (CFA) to validate the model fit of the measurement. The data analysis procedure and criteria are introduced in Appendix A.

#### Main study

A two-level linear model was used to test the main research questions and control for non-independence in the data due to provincial-level clusters of individual-level tennis officials. Given tennis officials primarily work under the administration of provincial-level tennis governing body, recognizing the individual–provincial hierarchy in the data was appropriate and necessary. Specifically, a series of models were specified with tennis officials nested within 26 provinces, beginning with an intercept-only model (M0) to evaluate the intraclass correlation (ICC). For ease of interpretation of model results, age, gender, officiating levels, SES, and event hosted were effect coded, and psychometrical measured variables were standardized as *z*-scores, with level 1 predictors cluster-mean centered and level 2 predictors grand-mean centered (see Table 1). The final model (M3) with random intercept was as follows:


$$\begin{array}{c} \mathrm{Communit}y_{ij}=\;\gamma_{0.0}\;+\;\gamma_{1.0}*\mathrm{FemEf}f_{i.j}\;+\;\gamma_{0.1}*L2Fem_{1.j}\;+\;\gamma_{2.0}*\mathrm{Off}W_{i.j}\;\\ +\;\gamma_{0.2}*L2\mathrm{Off}W_{2.j}\;+\;\gamma_{3.0}*\mathrm{Off}N_{i.j}+\;\gamma_{0.3}\;*L2\mathrm{Off}N_{3.j}\;\\ +\;\gamma_{4.0}*\mathrm{AgeEf}f_{i.j}+\;\gamma_{0.4}*L2Age_{4.j}\;+\;\gamma_{5.0}*\mathrm{SESHig}h_{i.j}\;\\ +\;\gamma_{0.5}*L2\mathrm{SESHig}h_{5.j}\;+\;\gamma_{6.0}*\mathrm{SESLo}w_{i.j}\\ +\;\gamma_{0.6}*L2\mathrm{SESLo}w_{6.j}\;+\;\gamma_{0.7}*\mathrm{EventEf}f_{7.j}\;\\ +\;\gamma_{7.0}*\mathrm{Admi}n_{i.j}\;+\;\gamma_{0.8}*L2\mathrm{Admi}n_{8.j}\;+\;\gamma_{8.0}*\mathrm{Stres}s_{i.j}\;\\ +\;\gamma_{0.9}*L2\mathrm{Stres}s_{9.j}\;+\;\gamma_{9.0}*\mathrm{ConEducatio}n_{i.j}\;\\ +\;\gamma_{0.10}*L2\mathrm{ConEducatio}n_{10.j}\;+\;\gamma_{10.0}*\mathrm{Mentorin}g_{i.j}\;\\ +\;\gamma_{0.11}*L2\mathrm{Mentorin}g_{11.j}\;+\;\gamma_{11.0}*\mathrm{Remuneratio}n_{i.j}\;\\ +\;\gamma_{0.12}*L2\mathrm{Remuneratio}n_{12.j}\;+\;\gamma_{12.11}*L1\mathrm{Mentorin}g_{i.j}\\ {}*L2\mathrm{Mentorin}g_{11.j}\;+\;\gamma_{0.13}*L2\mathrm{Admi}n_{8.j}\;\\ {}*\mathrm{Event}\_Eff_{7.j}\;+\;U_{0.j}\;+\;r_{i.j} \end{array}$$

In the model above, the *i*^th^ perceived sense of community of the *j*^th^ provinces is equal to the sum of the conditional mean ( $\gamma_{0.0}$ ), the unique effects of female status, official levels, age, SES, administrator consideration, stress, continuing education, mentoring, remuneration, two interactions ( $\gamma_{1.0}-\gamma_{0.13}$ ), and the residual error due to provincial membership ( $U_{0.j}$ ), and tennis officials ( $r_{i.j}$ ).


$$\begin{array}{c} \mathrm{Motivatio}n_{ij}\;=\;\gamma_{0.0}\;+\;\gamma_{1.0}*\mathrm{FemEf}f_{i,j}\;+\;\gamma_{0.1}*L2Fem_{1,j}\;+\;\gamma_{2.0}*\mathrm{Off}W_{i,j}\;\\ +\;\gamma_{0.2}*L2\mathrm{Off}W_{2,j}\;+\;\gamma_{3.0}*\mathrm{Off}N_{i,j}+\;\gamma_{0.3}*L2\mathrm{Off}N_{3,j}\;\\ +\;\gamma_{4.0}*\mathrm{AgeEf}f_{i,j}+\;\gamma_{0.4}*L2Age_{4,j}\;+\;\gamma_{5.0}*\mathrm{SESHig}h_{i,j}\\ +\;\gamma_{0.5}*L2\mathrm{SESHig}h_{5,j}\;+\;\gamma_{6.0}*\mathrm{SESLo}w_{i,j}\\ +\;\gamma_{0.6}*L2\mathrm{SESLo}w_{6,j}\;+\;\gamma_{0.7}*\mathrm{EventEf}f_{7,j}\;\\ +\;\gamma_{7.0}*\mathrm{Admi}n_{i,j}\;+\;\gamma_{0.8}*L2\mathrm{Admi}n_{8,j}\;\\ +\;\gamma_{8.0}*\mathrm{Stres}s_{i,j}\;+\;\gamma_{0.9}*L2\mathrm{Stres}s_{9,j}\;\\ +\;\gamma_{9.0}*\mathrm{ConEducatio}n_{i,j}\;+\;\gamma_{0.10}*L2\mathrm{ConEducatio}n_{10,j}\;\\ +\;\gamma_{10.0}*\mathrm{Mentorin}g_{i,j}\;+\;\gamma_{0.11}*L2\mathrm{Mentorin}g_{11,j}\;\\ +\;\gamma_{11.0}*\mathrm{Remuneratio}n_{i,j}\;+\;\gamma_{0.12}*L2\mathrm{Remuneratio}n_{12,j}\;\\ +\gamma_{12.0}*\mathrm{FemEf}f_{i,j}*\mathrm{Mentorin}g_{i,j}\;\\ +\;\gamma_{13.0}*\mathrm{ConEducatio}n_{i,j}*\mathrm{SESHig}h_{i,j}\;+\;U_{0,j}\;+\;r_{i,j} \end{array}$$

In the model above, the *i*^th^ motivation related to officiating of the *j*^th^ provinces is equal to the sum of the conditional mean ( $\gamma_{0.0}$ ), the unique effects of female status, official levels, age, SES, administrator consideration, stress, continuing education, mentoring, remuneration, two interactions ( $\gamma_{1.0}-\gamma_{13.0}$ ), and the residual error due to provincial membership ( $U_{0.j}$ ), and tennis officials ( $r_{i.j}$ ).

Effect sizes for fixed-effects coefficients were computed as approximate squared semi-partial correlation (*sr*^2^) values for each coefficient separately by dividing the coefficient by the product of the standard error and square root of the total sample size. Model *R*^2^ values for fixed and random effects were computed using *r2mlm* package (Rights and Sterba, 2019; Shaw et al., 2020); Model estimated with full information maximum likelihood were conducted using *lme4* and *lmerTest* packages (Bates et al., 2009; Kuznetsova et al., 2015). Data analyses were performed in R for Windows version 4.3.1 (R Core Team, 2021).

## Results

### Measurement validation and internal reliability

Seven latent variables were accepted as the most adequate structural representation of the 25-Item RRS-CN. Accordingly, CFAs were conducted on all latent variables, ensuring an adequate fit of the measurement model. Preliminary results from EFA and CFAs are shown in Appendix A. Given the results, the validated RRS-CN was then used in the main study for model testing.

### Main study

#### Correlations

Means, standard deviations (*SD*s), and zero-order correlations among all variables are given in Table 2. As can be seen, high SES, white badge or higher level officials, administrator consideration, stress, continuing education, and mentoring were significantly correlated with the sense of community, but they are also correlated with each other and may not uniquely predict the sense of community of participants related to officiating. Furthermore, besides the same predictors aforementioned except for stress, level 2 remuneration was also significantly correlated with motivation.

**Table 2:** Zero-order disaggregated correlations for variables used in analysis.

Measure	*ICC*	*M*	*(SD)*	1.		2.		3.		4.		5.		6.		7.		8.		9.		10.		11.		12.	
*Outcome*																											
1. Sense of community	0.03	6.46	(0.91)	–																							
2. Motivation	0.04	6.41	(0.90)	0.62	***	–																					
*Predictors*																											
3. Fem_Eff	0.02	0.27	0.45	−0.06		−0.05		–																			
4. L2_Fem	–	–	–	−0.02		−0.03		0.24	***	–																	
5. Off_W	0.16	0.06	0.24	−0.09	*	−0.07		−0.03		−0.08		–															
6. L2_Off_W	–	–	–	−0.11	*	−0.12	**	−0.04		−0.16	***	0.39	***	–													
7. Off_N	0.31	0.29	0.46	−0.04		−0.07		−0.09	*	−0.06		0.81	***	0.23	***	–											
8. L2_Off_N	–	–	–	−0.05		−0.04		−0.02		−0.09	*	0.47	***	0.26	***	0.56	***	–									
9. Age	0.07	0.44	0.50	−0.03		0.03		0.19	***	0.14	**	−0.49	***	−0.14	**	−0.48	***	−0.24	***	–							
10. L2_Age	–	–	–	0.06		0.07		0.10	*	0.40	***	−0.38	***	−0.38	***	−0.40	***	−0.69	***	0.36	***	–					
11. SES_H	0.06	0.15	0.36	0.00		0.02		−0.02		−0.02		0.17	***	0.08		0.14	**	0.07		−0.08		−0.05		–			
12. L2_SES_H	–	–	–	−0.11	**	−0.09	*	−0.05		−0.21	***	0.33	***	0.45	***	0.31	***	0.50	***	−0.21	***	−0.59	***	0.21	***	–	
13. SES_L	0.05	0.16	0.37	−0.02		0.02		0.04		0.07		−0.13	**	−0.04		−0.14	**	−0.09	*	0.30	***	0.18	***	0.72	***	−0.04	
14. L2_SES_L	–	–	–	0.04		0.06		0.07		0.31	***	−0.31	***	−0.35	***	−0.31	***	−0.53	***	0.30	***	0.84	***	−0.02		−0.59	***
15. Event	–	0.76	0.43	−0.06		0.01		0.01		0.06		−0.19	***	−0.17	***	−0.20	***	−0.35	***	0.10	*	0.27	***	0.03		−0.10	*
16. Admin	–	–	–	0.26	***	0.15	***	0.11	*	0.00		0.00		0.00		−0.02		0.00		−0.03		0.00		−0.05		0.00	
17. L2_Admin	0.20	4.91	1.69	0.04		−0.01		−0.02		−0.10	*	0.11	*	0.08		0.13	**	0.22	***	−0.03		−0.07		−0.03		0.01	
18. Stress	–	–	–	0.11	*	0.06		0.01		0.00		0.01		0.00		0.01		0.00		−0.14	**	0.00		−0.12	**	0.00	
19. L2_Stress	0.01	4.58	1.61	0.02		0.01		−0.04		−0.18	***	0.08		0.02		0.10	*	0.19	***	−0.05		−0.13	**	0.06		0.27	***
20. ConEducation	–	–	–	0.54	***	0.46	***	−0.06		0.00		−0.02		0.00		−0.04		0.00		−0.03		0.00		0.03		0.00	
21. L2_ConEducation	0.01	6.29	0.99	0.17	***	0.17	***	−0.05		−0.20	***	−0.15	***	−0.46	***	−0.06		−0.04		0.01		0.02		−0.03		−0.10	*
22. Mentoring	–	–	–	0.48	***	0.51	***	−0.06		0.00		−0.08		0.00		−0.09	*	0.00		0.04		0.00		−0.06		0.00	
23. L2_Mentoring	0.01	6.09	1.15	0.16	***	0.17	***	−0.05		−0.21	***	−0.22	***	−0.44	***	−0.17	***	−0.25	***	0.08		0.22	***	−0.04		−0.26	***
24. Remuneration	–	–	–	−0.03		−0.03		−0.03		0.00		−0.09	*	0.00		−0.11	*	0.00		0.24	***	0.00		−0.03		0.00	
25. L2_Remuneration	0.04	4.03	1.75	0.08		0.10	*	0.09	*	0.35	***	−0.25	***	−0.20	***	−0.28	***	−0.49	***	0.13	**	0.37	***	−0.06		−0.45	***

	13.		14.		15.		16.		17.		18.		19.		20.		21.		22.		23.		24.		25.		
*Outcome*																											
1. Sense of community																											
2. Motivation																											
*Predictors*																											
3. Fem_Eff																											
4. L2_Fem																											
5. Off_W																											
6. L2_Off_W																											
7. Off_N																											
8. L2_Off_N																											
9. Age																											
10. L2_Age																											
11. SES_H																											
12. L2_SES_H																											
13. SES_L	–																										
14. L2_SES_L	0.23	***	–																								
15. Event	0.11	*	0.38	***	–																						
16. Admin	−0.05		0.00		0.00		–																				
17. L2_Admin	−0.07		−0.21	***	−0.45	***	0.00		–																		
18. Stress	−0.16	***	0.00		0.00		0.51	***	0.00		–																
19. L2_Stress	0.00		−0.12	**	−0.28	***	0.00		0.52	***	0.00		–														
20. ConEducation	0.02		0.00		0.00		0.17	***	0.00		0.01		0.00		–												
21. L2_ConEducation	−0.02		−0.01		−0.09		0.00		0.06		0.00		0.26	***	0.00		–										
22. Mentoring	0.03		0.00		0.00		0.21	***	0.00		0.05		0.00		0.46	***	0.00		–								
23. L2_Mentoring	0.04		0.26	***	0.09	*	0.00		0.10	*	0.00		0.06		0.00		0.64	***	0.00		–						
24. Remuneration	0.06		0.00		0.00		−0.34	***	0.00		−0.40	***	0.00		0.05		0.00		0.11	*	0.00		–				
25. L2_Remuneration	0.08		0.47	***	0.24	***	0.00		−0.20	***	0.00		−0.18	***	0.00		0.11	*	0.00		0.45	***	0.00		–		

*N* = 523 participants within 26 provinces; Admin, administrator consideration; Stress, lack of stress; ConEducation, continuing education; Off_W, white-card or higher level officials; Off_N, national-level officials; Age, 35 years or younger (yrs); SES_H, high socioeconomic status; SES_Low, low socioeconomic status; Event, hosted one or more events annually (2019); ICCs were not reported in level 2 predictors; to note, categorical variables are dummy coded for mean and standard deviation (SD), and effect coded for Pearson’s rs; level 2 predictors were grand mean centered (GMC) and standardized in z scores that mean equals to zero and SD equals to one; ^*^p < 0.05, ^**^p < 0.01, ^***^p < 0.001.

#### Model results

The intercept-only model was specified to evaluate ICCs. As can be seen in Table 2, the first set of columns, the mean of the outcome variables ICCs, sense of community and motivation were 0.03 and 0.04, respectively, which were not significantly different from zero. The results indicated that 3% and 4% of variance in the sense of community and motivation scores, respectively, were explained by the provincial membership. The ICC for all the level 1 predictors ranged from 0.01 to 0.31, which was not significantly different from zero, indicating the expected correlation between any pair of random drawn predictor scores/categories within the same province is 0.01 to 0.31. Given the climate, ignoring non-zero dependencies in the outcomes and predictors by running a unlevel regression would cause biased slope standard errors and parameters, also known as blended slope problem (Raudenbush and Bryk, 2002; Snijders and Bosker, 2012). Therefore, a two-level modeling process was appropriate.

#### Sense of community

Next, demographic predictors were added to model 1. As shown in Table 3 (first set of columns), only the level 2 aggregate high SES (%) was significant. In the following, a group of predictors were added to model 2. As shown in Table 3 (second set of columns), officials with a white badge or higher, high or low SES, administrator consideration, mentoring, continuing education, level 2 aggregate high SES (%), and continuing education were significant. The approximate variance explained with this set of predictors was 0.44, which is 0.40 more than model 1. The likelihood ratio test (LRT) comparing model 2 to the previous, indicated a significant Chi-squared change (*χ*^2^ = 286.23, ∆*df* = 11, *p* < 0.001), and the Bayesian information criterion (BIC) value decreased by 217.40 points, indicating that the model with the second group of predictors was improving model-data fit.

**Table 3:** Multilevel linear model results for sense of community.

Fixed effects	Model 1						Model 2						Model 3					
	*Coeff*	*SE*	*t*	*df*		*ES*	*Coeff*	*SE*	*t*	*df*		*ES*	*Coeff*	*SE*	*t*	*df*		*ES*
Intercept (Mean)	6.30	0.08	83.47	523	***	12.83	6.31	0.06	98.22	523	***	10.27	6.34	0.06	97.96	523	***	9.98
1. Fem_Eff	−0.05	0.05	−1.08	523		0.00	−0.02	0.04	−0.60	523		0.00	−0.01	0.04	−0.42	523		0.00
2. L2_Fem	−0.08	0.08	−1.03	523		0.00	−0.02	0.08	−0.30	523		0.00	−0.01	0.08	−0.17	523		0.00
3. Off_W	−0.21	0.12	−1.75	523		0.01	−0.21	0.09	−2.31	523	*	0.01	−0.21	0.09	−2.29	523	*	0.01
4. L2_Off_W	0.03	0.08	0.43	523		0.00	0.07	0.08	0.85	523		0.00	0.03	0.08	0.43	523		0.00
5. Off_N	−0.05	0.08	−0.64	523		0.00	0.12	0.06	1.89	523		0.00	0.12	0.06	1.90	523		0.00
6. L2_Off_N	0.09	0.08	1.12	523		0.00	0.04	0.07	0.56	523		0.00	0.04	0.07	0.53	523		0.00
7. Age_Eff	−0.06	0.05	−1.08	523		0.00	−0.01	0.04	−0.22	523		0.00	−0.02	0.04	−0.37	523		0.00
8. L2_Age	0.15	0.14	1.12	523		0.00	0.13	0.13	1.04	523		0.00	0.15	0.13	1.14	523		0.00
9. SES_H	0.12	0.09	1.27	523		0.00	0.18	0.07	2.46	523	*	0.01	0.18	0.07	2.51	523	*	0.01
10. L2_SES_H	−0.10	0.09	−1.06	523	*	0.01	−0.16	0.07	−2.18	523	*	0.01	−0.14	0.07	−1.90	523		0.00
11. SES_L	−0.17	0.08	−2.14	523		0.00	−0.16	0.07	−2.20	523	*	0.01	−0.16	0.07	−2.30	523	*	0.01
12. L2_SES_L	−0.10	0.11	−0.86	523		0.00	−0.08	0.11	−0.68	523		0.00	−0.10	0.11	−0.93	523		0.00
13. Event							−0.06	0.04	−1.41	523		0.00	−0.04	0.04	−0.79	523		0.00
14. Admin							0.13	0.04	3.38	523	***	0.01	0.13	0.04	3.46	523	***	0.01
15. L2_Admin							−0.03	0.09	−0.38	523		0.00	−0.04	0.09	−0.46	523		0.00
16. Stress							0.01	0.04	0.31	523		0.00	0.01	0.04	0.40	523		0.00
17. L2_Stress							0.01	0.08	0.08	523		0.00	−0.03	0.08	−0.34	523		0.00
18. ConEducation							0.35	0.03	10.30	523	***	0.11	0.35	0.03	10.54	523	***	0.12
19. L2_ConEducation							0.17	0.08	2.01	523	*	0.00	0.20	0.08	2.34	523	*	0.01
20. Mentoring							0.27	0.03	7.65	523	***	0.06	0.26	0.03	7.49	523	***	0.06
21. L2_Mentoring							0.11	0.09	1.24	523		0.00	0.09	0.09	0.97	523		0.00
22. Remuneration							−0.01	0.03	−0.26	523		0.00	0.00	0.03	−0.13	523		0.00
23. L2_Remuneration							0.02	0.05	0.43	523		0.00	0.07	0.06	1.33	523		0.00
13. Event*15													0.19	0.08	2.56	523	*	0.01
20. Mentoring*21													−0.09	0.04	−2.41	523	*	0.01
**Random effects**	Var						*Var*						*Var*					
**Intercept (Provinces)**	< 0.01						< 0.01						< 0.01					
**Residual (Participants)**	0.80						0.46						0.45					
*Model fit*																		
Fixed-effects *R*^2^	0.04						0.44						0.46					
Random-effects *R*^2^	< 0.01						< 0.01						< 0.01					
BIC	1463.70						1246.30						1246.70					
Deviance (−2LL)	1369.80						1083.60						1071.40					
Residual *df*	508						497						495					
LRT Chi-square test	--						286.23	***					12.20	**				

*N* = 523 participants within 26 provinces; Admin, administrator consideration; Stress, lack of stress; ConEducation, continuing education; Off_W, white-card or higher levels officials; Off_N, national-level officials; Age, 35 years or younger (yrs); SES_H, high socioeconomic status; SES_Low, low socioeconomic status; ordinal predictors are cluster-mean centered (CMC) and standardized in z-scores and categorical predictors are effect coded (Eff); L2 refers to level 2 predictor; level 2 predictors are in standardized mean aggregate, grand-mean centered (GMC), in SDs; ES, effect size, reported as squared semi-partial correlation (sr^2^); model estimated with full information maximum likelihood using R lme4 and lmerTest packages. ^*^p < 0.05, ^**^p < 0.01, ^***^p < 0.001.

Afterward, two interaction terms were added to model 3. As shown in Table 3 (last set of columns), the approximate variance explained with this set of predictors was 0.46, which was 0.02 more than the previous model (model 2). The LRT comparing model 3 to the previous, indicated a significant chi-square change (*χ*^2^ = 12.20, ∆*df* = 2, *p* = 0.002), and the BIC value increased by 0.40 points, overall, indicating that the model with interactions was improving model-data fit.

In examining the coefficients for this final model, we see that the intercept, officials with white badge or higher, in high or low SES, administrator consideration, mentoring, continuing education, and level 2 aggregate continuing education were significant. Results indicated that the mean level of sense of community was 6.34 points, with all else held constant. Tennis officials with white badge or higher were predicted to be 0.21 points lower than the sample average on the sense of community, all else held constant. Tennis officials in high SES were predicted to be 0.18 higher than the sample average on the sense of community, all else held constant. Furthermore, for every *SD* increase in administrator consideration within their provinces, tennis officials’ sense of community was predicted to increase 0.13 points, all else held constant. For every *SD* increase in mentoring within their provinces, tennis officials’ sense of community was predicted to increase 0.26 points, all else held constant. In addition, for every *SD* increase in continuing education within their provinces, tennis officials’ sense of community was predicted to increase 0.35 points, all else held constant. Meanwhile, for every *SD* increase in provincial mean continuing education, tennis officials’ sense of community was predicted to increase 0.20 points, all else held constant.

Finally, two significant interactions were detected, one was between level 2 aggregate administrator consideration and event hosted, and the other was between mentoring and level 2 aggregate mentoring. To understand the nature of the interaction, model-implied values were computed for two levels of each (−1 *SD* and + 1 *SD*) and (+1 = hosted event, −1 = no event). As shown in Appendix B Figure 1, the first interaction was disordinal and showed that the positive relation between provincial mean administrator consideration (of 0.04 points per *SD* decreased administrator consideration) and sense of community was predicted to increase by 0.19 points for every *SD* increase if their provinces even hosted tennis events(s) in 2019. The other interaction was ordinal and showed that the positive relation between mentoring within their province (of 0.26 points per *SD* of increased mentoring) and sense of community was predicted to decrease by 0.09 points for every *SD* increase in their provincial mean mentoring (Appendix B Figure 2).

#### Motivation

Demographic predictors were first added to model 1. As shown in Table 4 (first set of columns), no predictor was found significant at the 0.05 level. In the following, the group of predictors was added to model 2. As shown in Table 4 (second set of columns), high SES, continuing education, mentoring, and remuneration were significant. The approximate variance explained with this set of predictors was 0.39, which is 0.37 more than model 1. The LRT comparing model 2 to the previous model indicated a significant chi-square change (*χ*^2^ = 243.26, ∆*df* = 11, *p* < 0.001), and the BIC value decreased by 243.30 points, indicating that the model with the second group of predictors was improving model-data fit.

**Table 4:** Multilevel linear model results for motivation.

*Fixed effects*	Model 1						Model 2						Model 3					
	*Coeff*	*SE*	*t*	*df*		*ES*	*Coeff*	*SE*	*t*	*df*		*ES*	*Coeff*	*SE*	*t*	*df*		*ES*
Intercept (Mean)	6.38	0.08	76.99	65	***	87.23	6.42	0.07	87.43	84	***	55.53	6.40	0.07	87.55	88	***	51.50
1. Fem_Eff	−0.06	0.05	−1.28	499		0.00	−0.02	0.04	−0.42	503		0.00	−0.02	0.04	−0.68	505		0.00
2. L2_Fem	−0.08	0.09	−0.87	36		0.02	−0.10	0.10	−1.00	44		0.01	−0.11	0.10	−1.09	47		0.02
3. Off_W	0.04	0.12	0.37	499		0.00	0.06	0.09	0.66	503		0.00	0.04	0.09	0.41	505		0.00
4. L2_Off_W	−0.16	0.09	−1.73	42		0.07	−0.07	0.09	−0.72	42		0.01	−0.03	0.09	−0.36	44		0.00
5. Off_N	−0.09	0.08	−1.17	499		0.00	−0.03	0.06	−0.45	503		0.00	−0.02	0.06	−0.36	504		0.00
6. L2_Off_N	0.11	0.09	1.14	28		0.04	0.16	0.09	1.77	34		0.06	0.16	0.09	1.80	35		0.05
7. Age_Eff	0.01	0.05	0.25	499		0.00	0.07	0.04	1.60	503		0.00	0.06	0.04	1.43	504		0.00
8. L2_Age	0.10	0.16	0.61	37		0.01	0.24	0.15	1.57	47		0.03	0.26	0.15	1.70	49		0.03
9. SES_H	0.10	0.09	1.15	499		0.00	0.18	0.07	2.45	503	*	0.01	0.17	0.07	2.38	505	*	0.01
10. L2_SES_H	−0.06	0.09	−0.67	36		0.01	−0.08	0.09	−0.92	53		0.01	−0.09	0.09	−1.01	56		0.01
11. SES_L	−0.07	0.09	−0.72	499		0.00	−0.14	0.07	−1.95	503		0.00	−0.13	0.07	−1.76	505		0.00
12. L2_SES_L	−0.07	0.13	−0.53	45		0.01	−0.18	0.13	−1.32	46		0.02	−0.18	0.13	−1.36	48		0.02
13. Event							−0.01	0.06	−0.20	19		0.00	−0.01	0.06	−0.23	20		0.00
14. Admin							−0.01	0.04	−0.33	503		0.00	−0.01	0.04	−0.33	505		0.00
15. L2_Admin							−0.11	0.11	−0.94	24		0.02	−0.09	0.11	−0.78	25		0.01
16. Stress							0.01	0.04	0.38	503		0.00	0.01	0.04	0.30	504		0.00
17. L2_Stress							0.07	0.11	0.65	31		0.01	0.04	0.11	0.40	32		0.00
18. ConEducation							0.25	0.03	7.25	503	***	0.06	0.31	0.04	7.16	516	***	0.06
19. L2_ConEducation							0.09	0.11	0.80	33		0.01	0.12	0.11	1.11	34		0.02
20. Mentoring							0.37	0.04	10.38	503	***	0.13	0.32	0.04	8.19	507	***	0.08
21. L2_Mentoring							0.11	0.12	0.91	27		0.02	0.08	0.12	0.70	29		0.01
22. Remuneration							−0.08	0.04	−2.25	503	*	0.01	−0.08	0.03	−2.28	504	*	0.01
23. L2_Remuneration							0.11	0.07	1.54	24		0.06	0.11	0.07	1.65	26		0.06
1. Fem_Eff*20													−0.08	0.04	−2.25	515	*	0.01
9. SES_H*18													0.11	0.04	2.48	523	*	0.01
**Random effects**	*Var*						*Var*						*Var*					
**Intercept (Provinces)**	0.02						0.01						0.01					
**Residual (Participants)**	0.77						0.48						0.47					
*Model fit*																		
Fixed-effects *R*^2^	0.02						0.39						0.40					
Random-effects *R*^2^	< 0.01						< 0.01						< 0.01					
BIC	1449.80						1275.50						1276.00					
Deviance (−2LL)	1356.00						1112.70						1100.80					
Residual *df*	508						497						493					
LRT Chi-square test	--						243.26	***					11.92	**				

*N* = 523 participants within 26 provinces; Admin, administrator consideration; Stress, lack of stress; ConEdeucation, continuing education; Off_W, white-card or higher levels officials; Off_N, national-level officials; Age, 35 years or younger (yrs); SES_H, high socioeconomic status; SES_Low, low socioeconomic status; ordinal predictors are cluster-mean centered (CMC) and standardized in z-scores and categorical predictors are effect coded (Eff); L2 refers to level 2 predictor; level 2 predictors are in standardized mean aggregate, grand-mean centered (GMC), in SDs; ES, effect size, reported as squared semi-partial correlation (sr^2^); model estimated with full information maximum likelihood using R lme4 and lmerTest packages. ^*^p < 0.05, ^**^p < 0.01, ^***^p < 0.001.

Afterward, two interaction terms were added to model 3. As shown in Table 4 (last set of columns), the approximate variance explained with this set of predictors was 0.40, which is 0.01 more than the previous model. The LRT comparing model 3 to the previous model indicated a significant chi-squared change (*χ*^2^ = 11.92, ∆*df* = 4, *p* = 0.003), and the BIC value decreased by 11.90 points, indicating that the model with interactions was improving model-data fit.

Examining the coefficients for this final model, we see that the intercept, high SES, continuing education, mentoring, and remuneration were significant. Results indicated that the mean level of motivation was 6.40 points, all else held constant. Tennis officials with high SES were predicted to be 0.17 higher than the sample average on motivation, all else held constant. Furthermore, for every *SD* increase in continuing education within their provinces, motivation of officiating was predicted to increase by 0.31 points, all else held constant. In addition, for every *SD* increase in mentoring within their provinces, motivation of officiating was predicted to increase 0.32 points, all else held constant. Furthermore, for every *SD* increase in remuneration within their provinces, motivation of officiating was predicted to decrease 0.08 points, all else held constant.

Ultimately, two significant interactions were detected, one was between female status and mentoring, and the other was between high SES and continuing education. To understand the nature of the interaction, model-implied values were computed for two levels of each (+1 = female, −1 = male) and (1 = SES high, 2 = medium, 3 = low). As shown in Appendix B Figure 3, the first interaction was disordinal and showed that the positive relation between mentoring within their provinces (of 0.32 points per *SD* of increased mentoring) and motivation of officiating was predicted to decrease by 0.08 points for every *SD* increase as being female officials. In addition, the other interaction was ordinal and showed that the positive relation between continuing education within their province (of 0.31 points per *SD* of increased continuing education) and motivation of officiating was predicted to increase by 0.11 points in high SES.

## Discussion

In the current study, we aimed to validate the RRS-CN measurement tool and use it to investigate the relationship between tennis officials’ individual characteristics, job retention factors, and their sense of community and motivation. A total of 523 tennis officials, representing a broad and geographically diverse sample across China, fully completed the survey. Using RRS-CN, we discovered that tennis officials’ sense of community and motivation are influenced by diverse individual and macro, provincial-level predictors.

A strong sense of community is an essential aspect of job satisfaction and is directly linked to retention (Warner et al., 2013; Kim et al., 2022). Chinese tennis officials, especially those in high SES, exhibited a greater sense of community within officiating. Our findings align with previous research showing that administrator fairness, transparency in game assignments, and networking contribute to officials’ retention (Ridinger, 2015; Kim, 2016; Ridinger et al., 2017). Several researchers emphasized the importance of referees’ feeling a sense of community to improve their engagement and psychological wellbeing (Livingston et al., 2017; Kim et al., 2022). However, high-level Chinese tennis officials reported a lower sense of community than their lower level counterparts, possibly due to social isolation and a lack of organizational understanding of mental health vulnerability. This empirical evidence provides new insights on the elite group’s need for greater recognition and belonging. Future research is needed to investigate mechanisms to enhance feelings of belonging and emotional connection (e.g., increased roles within the community) and the impact of these mechanisms on a sense of community and an individual’s wellbeing. Moreover, officials who were underpaid were more motivated to fulfill their duties, with remuneration negatively impacting their motivation to officiate. This could be attributed to tennis official’s high and middle socioeconomic status, making them less focused on monetary rewards. The role of a tennis official serves as a symbol of their unique middle-class lifestyle (Gong, 2020).

Establishing stable mentoring relationships and offering continuous education opportunities can motivate individuals to pursue officiating positions and strengthen community bonds (Kim and Hong, 2016; Nordstrom et al., 2016; Schaeperkoetter, 2016; Ridinger et al., 2017). Sport remains a highly masculinized space where women are underrepresented in officiating and leadership positions. Toxic and male-dominated sport environments are negatively affecting female officials’ wellbeing, motivation, and officiating persistence (Schaeperkoetter, 2016; Livingston et al., 2017; Webb et al., 2021; Tingle et al., 2022). Similarly, our findings show that quality mentorship is a contributor to motivation; however, female tennis officials were found less motivated compared to their male counterparts. Tsang and Lanusi (2022) introduced a women-to-women mentorship program in helping to bring long-term support and dynamic learning for female mentees. In this way, we suggest that future research could utilize egocentric network analysis as a potential method to delve deeper into understanding the needs and expectations of female officials in mentoring relationships and programs. Such insights could significantly enhance the recruitment, retention, and training of female tennis officials, ultimately benefiting the entire officiating community.

Furthermore, the Chinese Tennis Association has organized numerous training programs and workshops in the past decade to develop tennis officials (Xinhua News Agency, 2016). This has fostered a motivated community of officials who are committed to officiating for years to come. Interestingly, stress did not significantly impact the sense of community or motivation in this study, which has been previously linked to job satisfaction and retention (Anshel et al., 2013; Warner et al., 2013). According to Haugen (2020) and Rick and Li (2023), tennis is considered a “high-end” and “elegant” sport in China, and its unique game culture differentiates it from other sports, potentially influencing officiating dynamics.

The current research does possess several limitations. First, the generalizability of study results is limited due to the survey design and sampling technique that participants represented a convenience sample from China. Furthermore, the pre-pandemic data might not accurately reflect current job satisfaction levels among tennis officials. Although the current study can serve as a pre-pandemic reference point for future research, its value may be limited. Additionally, the scale validation process is also a limitation because predictive and concurrent validities of RRS-CN were not evaluated. Finally, the two-level model employed in this study, with individual tennis officials nested at the provincial level, does not account for possible systematic missingness and partial nesting. Future research should develop a more ecologically valid model, incorporating factors such as officiating experience, city clusters, mentoring relationships, and continuing education opportunities into the analysis.

## Data availability statement

The raw data supporting the conclusions of this article will be made available by the authors, without undue reservation.

## Ethics statement

The studies involving humans were approved by Hebei Institute of Physical Education Institutional Review Board. The studies were conducted in accordance with the local legislation and institutional requirements. The participants provided their written informed consent to participate in this study.

## Author contributions

LL: conceptualization, methodology, data analysis, processed the data, and wrote the manuscript. YL: data collection, manuscript review, and project administration. All authors contributed to the article and approved the submitted version.

## Footnotes

^1^According to International Tennis Federation (2023), there are four levels of officiating: (1) National; (2) Level 1, denoted as ITF Green Badge officials; (3) Level 2, known as White Badge; (4) Level 3, which includes Bronze, Silver, and Gold Badges.
