# Supplementary material for: Community and motivation among tennis officials: a cross-cultural multilevel analysis
Source: Front Psychol. 2023 Dec 19;14:1238153. doi: 10.3389/fpsyg.2023.1238153 (PMC10762873; doi:10.3389/fpsyg.2023.1238153)
Supplement: Supplementary file 1 [file Data_Sheet_1.docx]

**Appendix A**

**Exploratory and Confirmatory Factor Analysis**

Firstly, an exploratory factor analysis (EFA) was used to provide initial internal structural evidence for the RRS-CN, based on the data from pilot study (*n* = 180, see Appendix Table 1). Bartlett’s test of sphericity was used to ensure that the correlation matrix was not random, and the Kaiser–Meyer–Olkin (KMO) statistic was required to be above a minimum of .50. After confirming the correlation matrix was factorable, items were submitted for EFA. In EFA, an iterated principal axis extraction method with initial communalities estimated by squared multiple correlations was employed. To determine the appropriate number of factors to retain, parallel analysis and the visual scree test were applied (Velicer et al., 2000). Parsimony and theoretical convergence were also considered. The oblimin rotation method was employed because of the nature of the constructs that factors would be correlated. Criteria for determining factor adequacy were established a priori. Given the number of participants in this study, pattern coefficients ≥.37 were considered salient (Watkins, 2018). Complex loadings that were salient on more than one factor were rejected to honor simple structure, and factors with a minimum of two salient pattern coefficients, and that were theoretically meaningful were considered adequate (Watkins, 2018).

Univariate skewness and kurtosis were extreme based on the nature of our survey questions. Mardia’s multivariate skew and kurtosis were both statistically significant (*p* < .001). Given this nonnormality and the ordinal nature of the items, a polychoric correlation matrix is deemed an appropriate input for the EFA. Results of Bartlett’s test of sphericity indicated that the correlation matrix was not random, *χ*^2^(28) = 3265.86, *p* < .001, and the KMO test was .85, exceeding the threshold for conducting factor analysis. Given the results of the parallel analysis and the scree-plot, the assumption of a seven-factor structure was retained. Parallel analysis exhibited that seven factors were appropriate: administrator consideration, mentoring, continuing education, lack of stress, remuneration, sense of community, and intrinsic motives. The result was also in line with the scree-plot. To note, continuing education, including three items V21, V22 and V23, showed negative factor loadings by using the direct oblimin rotation method. Considering aforementioned items were not reversely coded, the promax rotation method was then employed to further confirm the factor structure. Similar factor loadings regarding all factors were observed; Specially, items, V21, V22 and V23, were found positively loaded on continuing education. Therefore, the initial seven-factor structure was retained due to its practical and theoretical appropriateness. Three items, V08, V24, and V26, were removed from the RRS-CN because factor loadings were not suppressing the threshold (Watkins, 2018). Given these results, the seven-factor solution was accepted as the most adequate structural representation of the 25-Item RRS-CN to empirical data was observed and was subsequently found to be robust across alternative extraction and rotation methods. Cronbach alpha ranged from .72 to .92. Descriptive statistics and translated measured items in RRS-CN are illustrated in Appendix A Table 2 and EFA results is presented in Appendix Table 3 and 4. Variance and covariance of emerged factors is listed in Appendix Table 5.

To further validate the model fit of the measurements, we conducted a Confirmatory Factor Analysis (CFA), based on the second wave of data (*n* = 343). The goodness of fit test was carried out on the basis of several indices. Chi-squared: non-significant values associated with *p* indicate a good model fit. To determine the adequacy of fit of the models to the data, the main index of fit examined was the comparative fit index (CFI). The CFI reflects model fit relatively well with all sample sizes, and ranges from 0 to 1, with values over .90 being required for a good or desirable fit, and preferably over .95 (Rupp & Segal, 1989). Tucker-Lewis Index (TLI): values higher than 0.90 indicate an acceptable fit. Root Mean Square Error of Approximation (RMSEA) and Standardized Root Mean Square Residual (SRMR): values less than .05 are desired, although less than .10 or .08 indicate an acceptable model fit (Hu & Bentler, 1998; Schmider et al., 2010; Zurita Ortega et al., 2018). The factor structures of the administrator consideration, mentoring, continuing education, lack of stress, remuneration, sense of community, and intrinsic motives were tested using maximum likelihood (ML) method. A summary of model fit statistics is presented in the following.

Regarding the model fit of administrator consideration, the chi-square test statistic was significant at .05 (*χ*^2^ = 584.15, *df* = 3, *p* < .001), which suggests the model is not perfectly fit, but it is acceptable. In factor analysis, chi-square statistics has no upper limit which cannot be interpreted in a standardized way and may also be sensitive to sample size (Zurita Ortega et al., 2018). CFI was 1.00 and TLI was 1.00, which indicates an excellent model fit. Finally, RMSEA and SRMR were zero and zero, respectively, which were less than 0.06 and indicate an excellent model fit. In general, an excellent fit of the model to empirical data was observed.

Regarding the model fit of intrinsic motives, the chi-square test statistic was significant at .05 (*χ*^2^ = 98.27, *df* = 9, *p* < .001), which suggests the model is not perfectly fit, but it is acceptable. In factor analysis, chi-square statistics has no upper limit which cannot be interpreted in a standardized way and may also be sensitive to sample size (Zurita Ortega et al., 2018). CFI was .95 and TLI was .92, which indicates an excellent model fit. Finally, RMSEA was .14 and SRMR was less than 0.06 and indicate an acceptable model fit. In general, a good fit of the model to empirical data was observed.

Regarding the model fit of mentoring, the chi-square test statistic was not significant at .05 (*χ*^2^ = 5.40, *df* = 2, *p* = .067), which suggests the model is perfectly fit. CFI was 1.00 and TLI was .99, which indicates an excellent model fit. Finally, RMSEA and SRMR were .06 and .01, respectively, which were equal and/or less than 0.06 and indicate an excellent model fit. In general, an excellent fit of the model to empirical data was observed.

Regarding the model fit of remuneration, the chi-square test statistic was significant at .05 (*χ*^2^ = 851.95, *df* = 3, *p* < .001), which suggests the model is not perfectly fit, but it is acceptable. In factor analysis, chi-square statistics has no upper limit which cannot be interpreted in a standardized way and may also be sensitive to sample size (Zurita Ortega et al., 2018). CFI was 1.00 and TLI was 1.00, which indicates an excellent model fit. Finally, RMSEA and SRMR were zero and zero, respectively, which were less than 0.06 and indicate an excellent model fit. In general, an excellent fit of the model to empirical data was observed.

Regarding the model fit of sense of community, the chi-square test statistic was significant at .05 (*χ*^2^ = 785.11, *df* = 3, *p* < .001), which suggests the model is not perfectly fit, but it is acceptable. In factor analysis, chi-square statistics has no upper limit which cannot be interpreted in a standardized way and may also be sensitive to sample size (Zurita Ortega et al., 2018). CFI was 1.00 and TLI was 1.00, which indicates an excellent model fit. Finally, RMSEA and SRMR were zero and zero, respectively, which were less than 0.06 and indicate an excellent model fit. In general, an excellent fit of the model to empirical data was observed.

Regarding the model fit of lack of stress, the chi-square test statistic was significant at .05 (*χ*^2^ = 725.26, *df* = 3, *p* < .001), which suggests the model is not perfectly fit, but it is acceptable. In factor analysis, chi-square statistics has no upper limit which cannot be interpreted in a standardized way and may also be sensitive to sample size (Zurita Ortega et al., 2018). CFI was 1.00 and TLI was 1.00, which indicates an excellent model fit. Finally, RMSEA and SRMR were zero and zero, respectively, which were less than 0.06 and indicate an excellent model fit. In general, an excellent fit of the model to empirical data was observed.

Regarding the model fit of continuing education, the chi-square test statistic was significant at .05 (*χ*^2^ = 680.21, *df* = 3, *p* < .001), which suggests the model is not perfectly fit, but it is acceptable. In factor analysis, chi-square statistics has no upper limit which cannot be interpreted in a standardized way and may also be sensitive to sample size (Zurita Ortega et al., 2018). CFI was 1.00 and TLI was 1.00, which indicates an excellent model fit. Finally, RMSEA and SRMR were zero and zero, respectively, which were less than 0.06 and indicate an excellent model fit. In general, an excellent fit of the model to empirical data was observed.

Regarding the model fit of the overall model, 25-Item RRS-CN, the chi-square test statistic was significant at .05 (*χ*^2^ = 647.37, *df* = 254, *p* < .001), which suggests the model is not perfectly fit, but it is acceptable. In factor analysis, chi-square statistics has no upper limit which cannot be interpreted in a standardized way and may also be sensitive to sample size (Zurita Ortega et al., 2018). CFI was .95 and TLI was .94, which indicates an excellent model fit. Finally, RMSEA and SRMR were .05 and .05, respectively, which were less than 0.06 and indicate an excellent model fit. In general, an excellent fit of the model to empirical data was observed.

**References**

Hu, L. T., & Bentler, P. M. (1998). Fit indices in covariance structure modeling: Sensitivity to underparameterized model misspecification. *Psychological Methods, 3*(4), 424–453. https://doi.org/10.1037/1082-989X.3.4.424

Rupp, M. T., & Segal, R. (1989). Confirmatory factor analysis of a professionalism scale in pharmacy. *Journal of Social and Administrative Pharmacy, 6*(1), 31–38.

Schmider, E., Ziegler, M., Danay, E., Beyer, L., & Bühner, M. (2010). Is it really robust? Reinvestigating the robustness of ANOVA against violations of the normal distribution assumption. *Methodology: European Journal of Research Methods for the Behavioral and Social Sciences, 6*(4), 147–151. https://doi.org/10.1027/1614-2241/a000016

Velicer, W. F., Eaton, C. A., & Fava, J. L. (2000). Construct explication through factor or component analysis: A review and evaluation of alternative procedures for determining the number of factors or components. In R. D. Goffin & E. Helmes (Eds.), *Problems and solutions in human assessment: Honoring Douglas N. Jackson at seventy* (pp. 41–71). Kluwer Academic/Plenum Press.

Watkins, M. W. (2018). Exploratory factor analysis: A guide to best practice. *Journal of Black Psychology, 44*(3), 219–246. https://doi.org/10.1177/0095798418771807

Zurita Ortega, F., Castro Sánchez, M., Chacón Cuberos, R., Cachón Zagalaz, J., Cofré Bolados, C., Knox, E., & Muros, J. J. (2018). Analysis of the psychometric properties of perceived motivational climate in sport questionnaire and its relationship to physical activity and gender using structural equation modelling. *Sustainability, 10*(3), 632. https://doi.org/10.3390/su10030632

| **Appendix A Table 1**  *Demographic Characteristics of Participants* | | | | |
| --- | --- | --- | --- | --- |
|  | Pilot Study (*n* = 180) | | Main Study (*N* = 523) | |
|  | Counts | % | Counts | % |
| Gender |  |  |  |  |
| Female | 48 | 26.67 | 143 | 27.34 |
| Male | 132 | 73.33 | 380 | 72.66 |
| Level of Officiating |  |  |  |  |
| White badge or higher | 13 | 7.22 | 32 | 6.12 |
| National Level | 73 | 40.56 | 154 | 29.45 |
| Level I | 80 | 44.44 | 266 | 50.86 |
| Level II or lower | 14 | 7.78 | 71 | 13.58 |
| Age |  |  |  |  |
| 20 or below | 24 | 13.33 | 6 | 1.15 |
| 21 - 30 years | 34 | 18.89 | 225 | 43.02 |
| 31 - 40 years | 66 | 36.67 | 172 | 32.89 |
| 41 or above | 56 | 31.11 | 120 | 22.94 |
| Education |  |  |  |  |
| Post-secondary certificate or lower | 9 | 5.00 | 24 | 4.59 |
| Bachelor’s degree | 69 | 38.33 | 257 | 49.14 |
| Master’s degree | 93 | 51.67 | 172 | 32.89 |
| Professional or Doctorate | 9 | 5.00 | 17 | 3.25 |
| Occupation |  |  |  |  |
| Student | 23 | 12.78 | 105 | 20.08 |
| Employed (Higher Education) | 82 | 45.56 | 202 | 38.62 |
| Employed (Enterprise and Public Institutions) | 55 | 30.56 | 135 | 25.81 |
| Others | 20 | 11.11 | 81 | 15.49 |
| Geographic Locations |  |  |  |  |
| North region | 90 | 50.00 | 136 | 26.00 |
| Eastern region | 82 | 45.56 | 89 | 17.02 |
| Central and Southern region | 5 | 2.78 | 173 | 33.08 |
| Western region | 3 | 1.67 | 125 | 23.90 |
| Note. Regarding to participants’ genders, there were no “Prefer Not to Answer “and “Non-Binary” had been reported by participants; Other occupation included self-employed, retired, and unable to work; According to the Chinese National Bureau of Statistics data released in 2011, geographic locations were summarized into four main regions. In the current study, North region included: Neimengu, Beijing, Tianjin, Hebei, Heilongjiang, Liaoning, and Jilin provinces; Eastern region included: Shanghai, Zhejiang, Fujian, Jiangsu; Central and Southern region included: Shandong, Henan, Jiangxi, Anhui, Hainan, Hubei, Hunan, and Guangdong; Western region: Chongqing, Guizhou, Yunnan, Shanxi, Guangxi, Xinjiang, Shanxi, and Sichuan; Lower category including elementary school, some high school, completed high school, associate degree, or other primary education experiences | | | | |

| **Appendix A Table 2**  *Descriptive Statistics for Items in RRS and RRS-CN* | | | | |
| --- | --- | --- | --- | --- |
| Item (Translated Items in Chinese) | Mean | *SD* | S | K |
| V01. I officiate as a way to stay involved with the sport.  (我将网球裁判看作是参与网球运动的一种方式) | 6.34 | 1.23 | -2.24 | -5.45 |
| V02. I enjoy officiating because it allows me to stay connected to my sport.  (我喜欢网球裁判工作是因为我能更好的与网球运动保持关联) | 6.25 | 1.44 | -2.23 | -4.55 |
| V03. Officiating allows me to give back to the sport.  (从事裁判工作能让我更好的回馈网球运动) | 6.40 | 1.23 | -2.43 | -6.92 |
| V04. I love the competitive nature of sports.  (我喜欢网球运动的竞技性) | 6.47 | 0.99 | -2.42 | -7.37 |
| V05. I like that officiating allows me to be part of competitive events.  (我喜欢执裁能使我成为竞赛的一部分) | 6.20 | 1.35 | -2.13 | -4.72 |
| V06. I like the challenge of officiating.  (我喜欢执裁给我带来的挑战) | 6.26 | 1.27 | -2.19 | -5.44 |
| V07. Officiating is a good source of supplementary income.  (参与裁判工作是赚取额外收入的一个渠道) | 4.48 | 1.89 | -0.29 | -0.83 |
| V08. Money is not the primary reason I officiate. R  (赚钱不是我做裁判的初衷) | 2.11 | 1.70 | -1.60 | -1.73 |
| V09. Pay was an important factor in my decision to start officiating.  (裁判工作收入是我开始执裁的一个重要因素) | 3.48 | 1.92 | -0.33 | -0.88 |
| V10. My main motivation for officiating is financial reward.  (物质奖酬是我做裁判的主要动力) | 3.67 | 1.88 | -0.17 | -0.91 |
| V11. A mentor helped me to feel welcomed in the officiating community.  (导师/前辈的帮助使我在裁判群体中更受欢迎) | 5.94 | 1.34 | -1.19 | -1.12 |
| V12. A mentor or friend encouraged me to officiate.  (导师/前辈或朋友鼓励我参与裁判工作) | 5.94 | 1.43 | -1.37 | -1.29 |
| V13. A mentor assisted my integration into the officiating community.  (导师/前辈协助我融入裁判群体) | 6.25 | 1.33 | -2.23 | -5.23 |
| V14. Having a mentor to support me as an official was an initial attraction to the role.  (初始吸引我从事裁判工作的原因是导师或前辈的支持) | 5.81 | 1.62 | -1.43 | -1.39 |
| V15. I often feel abuse while officiating.  (在执裁过程中，我经常感到疲惫不堪) R | 4.33 | 1.86 | -0.06 | -0.94 |
| V16. I often feel a lot of stress while officiating.  (在执裁过程中，我经常感到压力很大) R | 4.26 | 1.77 | -0.21 | -0.78 |
| V17. I often encounter hostile interactions with coaches and/or spectators while officiating.  (在执裁过程中，我经常陷入与教练员或是观众的紧张关系中) R | 4.92 | 1.83 | -0.64 | -0.52 |
| V18. I belong to a strong officiating community.  (我处在一个凝聚力较强的裁判群体中) | 6.27 | 1.25 | -2.14 | -4.85 |
| V19. I feel included in the officiating community.  (我觉得我是裁判队伍里的一员) | 6.48 | 1.03 | -2.83 | 10.00 |
| V20. A strong sense of community among officials exists for me.  (我认为裁判群体有较强的团队意识) | 6.32 | 1.28 | -2.34 | -5.67 |
| V21. Training prepared me for interactions with coaches, players, and fans.  (职业培训使我能够处理好教练，球员与球迷之间的关系) | 6.06 | 1.32 | -1.58 | -2.66 |
| V22. Because of the continuing education provided by my association, I feel prepared  to officiate my sport.  (通过参与由相关机构或协会提供的职业培训，使我做好了执裁的充分准备) | 6.43 | 0.96 | -2.08 | -5.88 |
| V23. I receive adequate training each year to stay current on officiating mechanics and  rules of the game.  (每年充分适当的职业培训使我在执裁技术与规则解读层面上与时俱进) | 6.38 | 1.12 | -2.31 | -6.30 |
| V24. Administrators in my officials’ association are considerate of my needs.  (在我所属的协会中，管理者能够考虑到我的需求) | 5.33 | 1.66 | -0.94 | -0.28 |
| V25. Officiating assignments are based on favoritism and politics.  (临场任务的指派主要靠权术与人际关系) R | 4.55 | 1.90 | -0.21 | -1.02 |
| V26. Decisions related to game assignments are fair.  (比赛任务的分配是公平的) | 5.75 | 1.48 | -1.25 | -1.13 |
| V27. Game assignments are distributed based on “who you know.”  (赛事技术官员的选派是由“你认识谁”决定的) R | 4.67 | 2.01 | -0.33 | -1.09 |
| V28. Administrators in my officials’ association show favoritism.  (我所属协会的管理者在工作中存在偏袒) R | 5.03 | 2.02 | -0.58 | -1.01 |
| *Note.* *n* = 180 participant; RRS-CN = Referee Retention Scale Chinese version; The English version of Referee Retention Scale (RRS) was extracted directly on p. 519 from Ridinger et al. (2017); R = item was reverse coded; “Sport” in V01-V04 was translated to “tennis” to be more specific and reflect the purpose of the current study; S = Skewness statistic ; K = Kurtosis statistic | | | | |

| **Appendix A Table 3**  *Communality and Pattern Coefficients for RRS-CN Items using Oblimin Rotation Method* | | | | | | | | |
| --- | --- | --- | --- | --- | --- | --- | --- | --- |
| Items | Factor Loading | | | | | | | *h*^2^ |
|  | Sense of Community | Lack of Stress | Administrator Consideration | Mentoring | Continuing Education | Remuneration | Intrinsic Motives |  |
| V20 | -**0.77** | -0.07 | -0.05 | -0.01 | -0.03 | -0.04 | -0.19 | .81 |
| V18 | -**0.74** | -0.03 | -0.13 | -0.11 | -0.01 | -0.02 | -0.17 | .84 |
| V19 | -**0.66** | -0.02 | -0.02 | -0.06 | -0.17 | -0.05 | -0.22 | .76 |
| V04 | -**0.37** | -0.03 | -0.01 | -0.11 | -0.16 | -0.05 | -0.35 | .42 |
| V24 | -0.34 | -0.16 | -0.19 | -0.24 | -0.05 | -0.10 | -0.10 | .32 |
| V16 | -0.00 | -**0.90** | -0.06 | -0.07 | -0.02 | -0.02 | -0.10 | .77 |
| V17 | -0.10 | -**0.84** | -0.10 | -0.05 | -0.04 | -0.07 | -0.06 | .83 |
| V15 | -0.05 | -**0.62** | -0.17 | -0.07 | 0.07 | -0.10 | -0.12 | .58 |
| V27 | -0.02 | -0.02 | -**0.75** | -0.04 | -0.06 | -0.15 | -0.01 | .67 |
| V25 | -0.08 | -0.19 | -**0.65** | -0.01 | -0.12 | -0.10 | -0.00 | .61 |
| V28 | -0.17 | -0.15 | -**0.62** | -0.08 | -0.06 | -0.03 | -0.03 | .55 |
| V26 | -0.22 | -0.08 | -0.36 | -0.21 | -0.14 | -0.03 | -0.02 | .38 |
| V11 | -0.02 | -0.00 | -0.02 | -**0.83** | -0.03 | -0.04 | -0.04 | .75 |
| V12 | -0.00 | -0.05 | -0.13 | -**0.79** | -0.00 | -0.06 | -0.13 | .77 |
| V14 | -0.13 | -0.03 | -0.04 | -**0.65** | -0.03 | -0.10 | -0.11 | .47 |
| V13 | -0.17 | -0.08 | -0.12 | -**0.61** | -0.06 | -0.10 | -0.09 | .61 |
| V23 | -0.09 | -0.02 | -0.09 | -0.04 | **-1.01** | -0.05 | -0.06 | .91 |
| V22 | -0.05 | -0.02 | -0.14 | -0.03 | **-0.73** | -0.07 | -0.07 | .68 |
| V21 | -0.03 | -0.07 | -0.13 | -0.08 | **-0.57** | -0.10 | -0.13 | .46 |
| V09 | -0.12 | -0.03 | -0.08 | -0.03 | -0.03 | -**0.90** | -0.03 | .81 |
| V10 | -0.05 | -0.07 | -0.05 | -0.10 | -0.01 | -**0.71** | -0.09 | .61 |
| V07 | -0.01 | -0.09 | -0.05 | -0.03 | -0.05 | -**0.65** | -0.01 | .49 |
| V08 | -0.21 | -0.01 | -0.18 | -0.18 | -0.19 | -0.22 | -0.07 | .23 |
| V05 | -0.04 | -0.02 | -0.00 | -0.05 | -0.06 | -0.00 | -**0.88** | .82 |
| V06 | -0.06 | -0.03 | -0.03 | -0.10 | -0.06 | -0.01 | -**0.83** | .77 |
| V03 | -0.13 | -0.05 | -0.02 | -0.17 | -0.03 | -0.11 | -**0.68** | .70 |
| V02 | -0.17 | -0.04 | -0.06 | -0.08 | -0.01 | -0.02 | -**0.67** | .62 |
| V01 | 0.20 | -0.09 | 0.00 | 0.07 | -0.12 | -0.16 | **0.55** | .63 |
| *α* | .92 | .87 | .75 | .86 | .82 | .72 | .91 |  |
| *Note*. *n* = 180 participants; RRS-CN = Referee Retention Scale Chinese version; *α* = Cronbach Alpha; *h*^2^ = Communality; Salient pattern coefficients ≥ .37 or ≤ -.37 in boldface. | | | | | | | | |

| **Appendix A Table 4**  *Communality and Pattern Coefficients for RRS-CN Items using Promax Rotation Method* | | | | | | | | |
| --- | --- | --- | --- | --- | --- | --- | --- | --- |
| Items | Factor Loading | | | | | | | *h*^2^ |
|  | Intrinsic Motives | Mentoring | Sense of Community | Administrator Consideration | Lack of Stress | Continuing Education | Remuneration |  |
| V05 | -**0.96** | -0.00 | -0.09 | -0.02 | -0.01 | -0.02 | -0.03 | .82 |
| V06 | -**0.90** | -0.06 | -0.12 | -0.04 | -0.02 | -0.03 | -0.02 | .77 |
| V03 | -**0.70** | -0.13 | -0.12 | -0.02 | -0.05 | -0.07 | -0.08 | .70 |
| V02 | -**0.70** | -0.03 | -0.16 | -0.07 | -0.05 | -0.06 | -0.04 | .62 |
| V01 | **-0.55** | -0.02 | -0.21 | -0.02 | -0.09 | -0.08 | -0.14 | .63 |
| V11 | -0.02 | **-0.88** | -0.01 | -0.04 | -0.01 | -0.00 | -0.05 | .75 |
| V12 | -0.09 | **-0.82** | -0.02 | -0.12 | -0.03 | -0.03 | -0.08 | .77 |
| V14 | -0.11 | **-0.68** | -0.17 | -0.04 | 0.03 | -0.02 | -0.10 | .47 |
| V13 | -0.03 | **-0.63** | -0.19 | -0.16 | -0.08 | -0.04 | -0.10 | .61 |
| V20 | -0.10 | -0.06 | **-0.91** | -0.01 | -0.06 | -0.05 | -0.07 | .81 |
| V18 | -0.07 | -0.04 | **-0.87** | -0.09 | -0.01 | -0.10 | -0.01 | .84 |
| V19 | -0.13 | -0.12 | **-0.78** | -0.02 | -0.01 | -0.10 | -0.03 | .76 |
| V04 | -0.33 | -0.16 | **-0.43** | -0.01 | -0.03 | -0.11 | -0.07 | .42 |
| V24 | -0.17 | -0.23 | -**0.41** | -0.18 | -0.18 | -0.01 | -0.11 | .32 |
| V08 | -0.14 | -0.19 | -0.27 | -0.23 | -0.00 | -0.18 | -0.24 | .23 |
| V27 | -0.01 | -0.06 | -0.01 | **-0.81** | -0.05 | -0.02 | -0.09 | .67 |
| V25 | -0.02 | -0.00 | -0.11 | **-0.70** | -0.14 | -0.10 | -0.05 | .61 |
| V28 | -0.05 | -0.05 | -0.19 | **-0.65** | -0.09 | -0.10 | -0.02 | .55 |
| V26 | -0.07 | -0.19 | -0.26 | -0.37 | -0.12 | -0.09 | -0.01 | .38 |
| V16 | -0.09 | -0.08 | -0.02 | -0.08 | **-0.92** | -0.03 | -0.04 | .77 |
| V17 | -0.06 | -0.06 | -0.10 | -0.08 | **-0.85** | -0.04 | -0.04 | .83 |
| V15 | -0.14 | -0.06 | -0.09 | -0.18 | **-0.62** | -0.07 | -0.07 | .58 |
| V23 | -0.06 | -0.03 | -0.07 | -0.07 | -0.02 | **-1.01** | -0.06 | .91 |
| V22 | -0.06 | -0.02 | -0.07 | -0.12 | -0.02 | **-0.71** | -0.09 | .68 |
| V21 | -0.13 | -0.07 | -0.05 | -0.16 | -0.09 | **-0.56** | -0.10 | .46 |
| V09 | -0.01 | -0.05 | -0.12 | -0.06 | -0.07 | -0.00 | -**0.92** | .81 |
| V10 | -0.06 | -0.11 | -0.07 | -0.03 | -0.05 | -0.00 | -**0.72** | .61 |
| V07 | -0.04 | -0.02 | 0.00 | -0.03 | -0.07 | -0.04 | **0.66** | .49 |
| *α* | .91 | .86 | .92 | .75 | .87 | .82 | .72 |  |
| *Note*. *n* = 180 participants; RRS-CN = Referee Retention Scale Chinese version; *α* = Cronbach Alpha; *h*^2^ = Communality; Salient pattern coefficients ≥ .37 or ≤ -.37 in boldface. | | | | | | | | |

| **Appendix A Table 5**  *Variance and Covariance Matrix of Factors* | | | | | | | | |
| --- | --- | --- | --- | --- | --- | --- | --- | --- |
| Factors | 1 | 2 | 3 | 4 | 5 | 6 | 7 |  |
| 1. Administrator Consideration | --1.66 |  |  |  |  |  |  |  |
| 2. Intrinsic Motives | -0.30 | --1.04 |  |  |  |  |  |  |
| 3. Mentoring | -0.53 | -0.69 | --1.45 |  |  |  |  |  |
| 4. Remuneration | -0.52 | -0.28 | -0.15 | -1.85 |  |  |  |  |
| 5. Sense of Community | -0.64 | -0.76 | -0.68 | -0.35 | -1.23 |  |  |  |
| 6. Lack of Stress | -0.79 | -0.08 | -0.23 | -0.94 | -0.20 | -2.62 |  |  |
| 7. Continuing Education | -0.38 | -0.48 | -0.52 | -0.14 | -0.55 | -0.06 | 0.95 |  |
| *Note*. *n* = 180 participants | | | | | | | | |
