# Supplementary material for: Community and motivation among tennis officials: a cross-cultural multilevel analysis
Source: Front Psychol. 2023 Dec 19;14:1238153. doi: 10.3389/fpsyg.2023.1238153 (PMC10762873; doi:10.3389/fpsyg.2023.1238153)
Supplement: Supplementary file 2 [file Data_Sheet_2.docx]

**Appendix B**

***
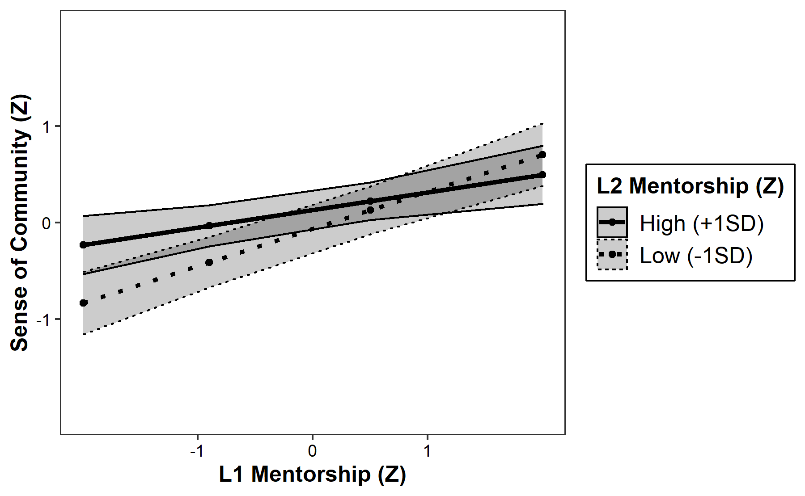
*Interaction Visualizations**

***Appendix B Figure 1.*** Predicted Sense of Community for Provincial level Administrator Consideration by Tennis Event(s) Hosted

***
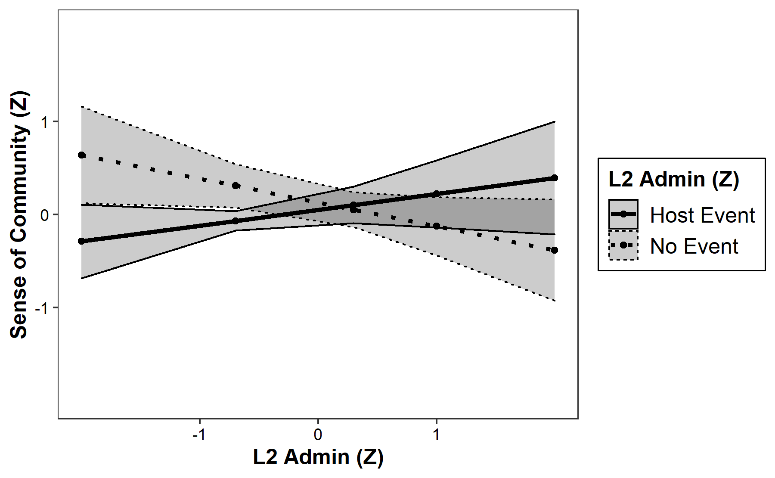
***

***Appendix B Figure 2.*** Predicted Sense of Community for Mentoring by Provincial level Mean Perceived Mentoring

***
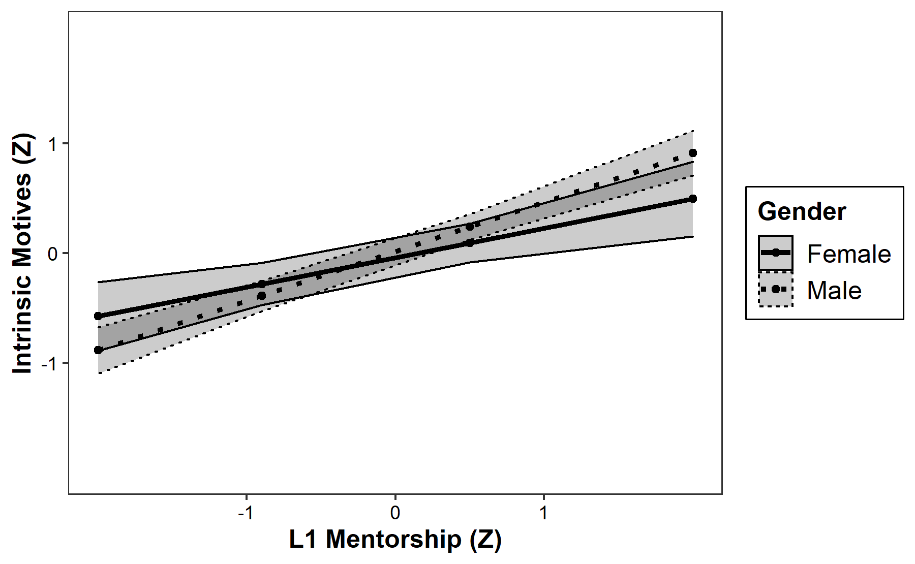
***

***Appendix B Figure 3.*** Predicted Motivation for Mentoring by Gender

***
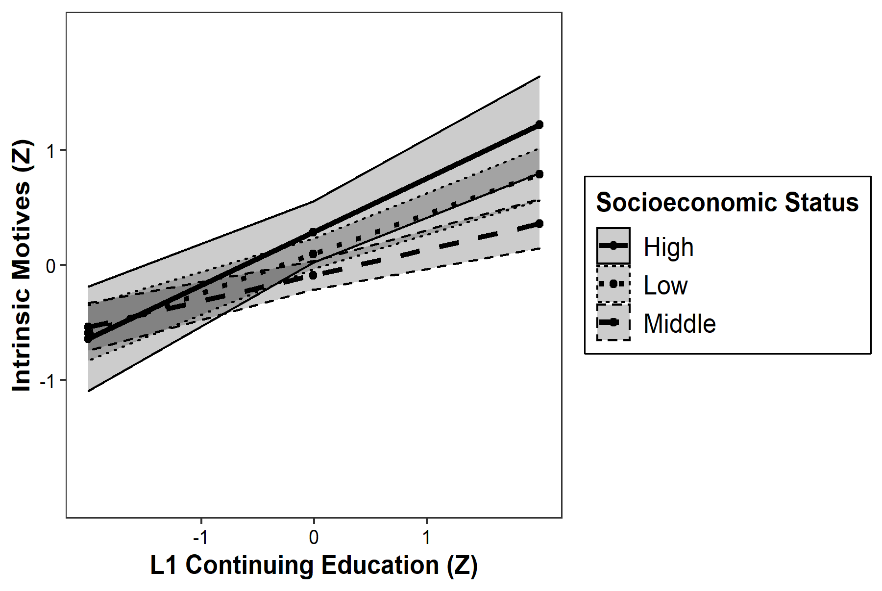
***

***Appendix B Figure 4.*** Predicted Motivation for Continuing Education by Socioeconomic Status
